# Extremely Narrow and Actively Tunable Mie Surface Lattice Resonances in GeSbTe Metasurfaces: Study

**DOI:** 10.3390/nano12040701

**Published:** 2022-02-20

**Authors:** Lei Xiong, Hongwei Ding, Yuanfu Lu, Guangyuan Li

**Affiliations:** 1 School of Information Science and Engineering, Yunnan University, Kunming 650500, China; lei.xiong@siat.ac.cn; 2 CAS Key Laboratory of Human-Machine Intelligence-Synergy Systems, Shenzhen Institute of Advanced Technology, Chinese Academy of Sciences, Shenzhen 518055, China; yf.lu@siat.ac.cn; 3 Shenzhen College of Advanced Technology, University of Chinese Academy of Sciences, Shenzhen 518055, China

**Keywords:** Mie surface lattice resonances, active tuning, GST metasurface, reconfigurable narrowband filter, all-optical modulation and switching

## Abstract

Mie surface lattice resonances (SLRs) supported by periodic all-dielectric nanoparticles emerge from the radiative coupling of localized Mie resonances in individual nanoparticles through Rayleigh anomaly diffraction. To date, it remains challenging to achieve narrow bandwidth and active tuning simultaneously. In this work, we report extremely narrow and actively tunable electric dipole SLRs (ED-SLRs) in Ge_2_Se_2_Te_5_ (GST) metasurfaces. Simulation results show that, under oblique incidence with TE polarization, ED-SLRs with extremely narrow linewidth down to 12 nm and high quality factor up to 409 can be excited in the mid-infrared regime. By varying the incidence angle, the ED-SLR can be tuned over an extremely large spectral region covering almost the entire mid-infrared regime. We further numerically show that, by changing the GST crystalline fraction, the ED-SLR can be actively tuned, leading to nonvolatile, reconfigurable, and narrowband filtering, all-optical multilevel modulation, or all-optical switching with high performance. We expect that this work will advance the engineering of Mie SLRs and will find intriguing applications in optical telecommunication, networks, and microsystems.

## 1. Introduction

Surface lattice resonances (SLRs), sometimes referred to as collective resonances or diffractively coupled localized resonances, were initially observed in periodically arranged plasmonic nanoparticles, owing to the coherent interference between the localized surface plasmon resonance (LSPR) and the Rayleigh anomaly (RA) diffracted light [1,2]. Because of their many appealing characteristics, such as large quality factors, strong field enhancements extended over large volumes, and large wavelength tunability, SLRs have emerged as a new platform for enhancing light-matter interactions on the nanoscale, and have found a diverse range of applications in sensing, optical filtering, structural color printing, fluorescence enhancement, nanoscale lasing, and nonlinear optics [1,2,3,4,5].

Recently, the scope of SLRs has further extended from lossy plasmonic nanoparticles to lossless all-dielectric nanoparticles with broadened applicability and enriched properties [2]. Evlyukhin et al. [6] theoretically investigated Mie SLRs in an array of silicon nanoparticles, and Tsoi et al. [7] performed experimental demonstration. Li et al. [8] numerically achieved sharp magnetic dipole SLR (MD-SLR) with sub-10 nm linewidth and quality factor of Q∼170. Here, the quality factor of the resonance *Q* is intrinsically linked to the ratio of energy stored to the energy lost by an oscillator, and can be estimated as evaluated as the ratio of the resonance wavelength to the linewidth. Castellanos et al. [9] systematically investigate MD-SLR and electric dipole SLR (ED-SLR) in dielectric nanoparticle arrays. The group of Rasskazov studied the effects of finite array size [10] and oblique incidence [11]. Babicheva and her colleagues investigated resonant lattice Kerker effect through spectrally overlapping ED-SLR and MD resonance of nanoparticles [12,13,14]. Recently, Murai et al. [15] experimentally demonstrated ED- and MD-SLRs, and obtained a high quality factor of Q=298 in experiments for the ED-SLR. However, these Mie SLRs are obtained in silicon nanoparticle array and thus cannot be actively tuned.

Phase-change materials, such as germanium-antimony-telluride (Ge_x_Sb_y_Te_z_, GST) [16], Sb [17], Sb_2_Te_3_ [18], Sb_2_S_3_, and Sb_2_Se_3_ [19,20], have shown to be a nonvolatile and reconfigurable platform for achieving dynamical tuning of various optical resonances. As the most commonly used material, GST provides nonvolatile, rapid, and reversible switching between the amorphous and the crystalline states by electrical or optical stimuli, significant refractive index differences between these two states, and high chemical and long-term stability [16,21,22,23]. Combining metal structures and GST films, enhanced plasmonic biosensors [24], tunable perfect absorption [25,26,27,28,29], plasmonic SLRs [30,31,32,33], or extraordinary optical transmission [34,35,36] were proposed or demonstrated, resulting in mid-infrared reflection bandstop or transmission bandpass filters, respectively. By embedding a GST film in a Fabry–Pérot (F–P) microcavity, tunable F–P resonance and bandpass filters were also proposed or demonstrated [37,38]. Based on GST metasurfaces, tunable Mie resonances [39,40,41,42,43,44] or Mie SLRs [45] were also investigated or demonstrated. However, all these GST-based tunable resonances show relatively low quality factors (usually less than 100). In other words, it remains challenging to simultaneously achieve high quality factor (or narrow bandwidth) and active tuning.

In this work, we provide a solution to this problem by launching ED-SLRs in a Ge_2_Se_2_Te_5_ nanorod array under oblique incidence with TE polarization. We will show that, by increasing the incidence angle from 0° to 70°, the resonance wavelength of the ED-SLR can be tuned over a very broad spectral region, which spans from 3.171 μm to 4.908 μm and covers almost the entire mid-infrared regime (3–5 μm). Meanwhile, the linewidth decreases dramatically to 12 nm, and the corresponding quality factor increases by more than ten times, from 38 to 409. We will also show that, by changing the GST crystalline fraction, which can be done using nanosecond [36] or femtosecond [42] optical pulses, the resonance wavelength of the ED-SLR can be dynamically tuned, enabling reconfigurable narrowband optical bandpass or bandstop filters, or multilevel all-optical modulation with large modulation depth, or even all-optical switching with high performance.

## 2. Design and Simulation Setups

**Figure 1:**
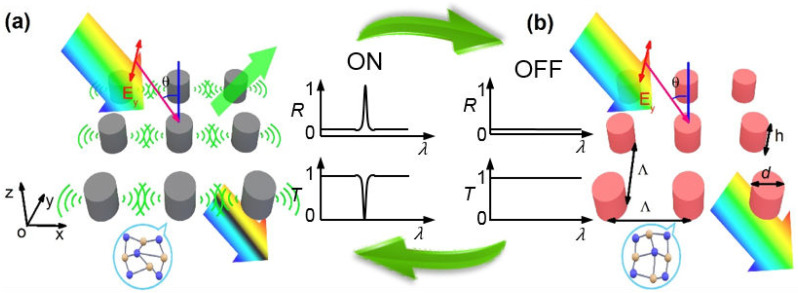
Schematics of the actively switchable ED-SLR in GST nanorod array under oblique incidence with TE polarization. (**a**) When GST is in the amorphous state, the ED-SLR is excited, resulting in extremely narrow bandpass filtering in reflection and bandstop filtering in transmission. (**b**) When GST is in a semi-crystalline state, the ED-SLR and the narrowband reflection/transmission filtering are switched off.

Figure 1 illustrates the GST metasurface under study. It is composed of periodic GST nanorods embedded in homogeneous dielectric environment of silica ($n_{0}=1.5$). The nanorods have period Λ=2 μm in the *x*- and *y*-directions, diameter d=0.8 μm, and height h=0.4 μm. Plane wave light impinges onto the metasurface at incidence angle θ.

The operation principle of the metasurface is designed as follows. When GST is in the amorphous state, the ED-SLR can be excited for the TE-polarization, leading to extremely narrow bandpass filtering in reflection and correspondingly bandstop filtering in transmission, as illustrated by Figure 1a. When GST is in a semi-crystalline state, however, Figure 1b illustrates that the ED-SLR as well as the resulted narrowband filtering is switched off.

The designed metasurface can be fabricated using the state-of-the-art nanofabrication processes following [42]. A GST film is first deposited on the silica substrate by magnetron sputtering, followed by spin coating of negative photoresist and subsequent e-beam exposure. After development, the pattern is transferred to the GST layer through reactive ion etching and photoresist removal. Finally, a thick silica film is deposited on the top, resulting in the designed GST nanorods embedded in silica.

The total reflectance and transmittance spectra, R(λ) and T(λ), as well the near-field distributions of the proposed metasurface were numerically simulated with a home-developed package for the fully vectorial rigorous coupled-wave analysis (RCWA), which was developed following [46,47,48]. In our simulations, we adopted retained orders of 41×41, which were shown to be large enough to reach the convergence regime. The dielectric constants of the GST material in different crystallization levels are determined by

(1) $$\begin{array}{c} \frac{\varepsilon_{\mathrm{eff}}\left(\lambda \right)-1}{\varepsilon_{\mathrm{eff}}\left(\lambda \right)+2}=m\frac{\varepsilon_{c}\left(\lambda \right)-1}{\varepsilon_{c}\left(\lambda \right)+2}+\left(1-m\right)\frac{\varepsilon_{a}\left(\lambda \right)-1}{\varepsilon_{a}\left(\lambda \right)+2}\;. \end{array}$$

Here, 0≤m≤1 is the GST crystalline fraction. $\varepsilon_{a}\left(\lambda \right)$ and $\varepsilon_{c}\left(\lambda \right)$, which are taken from [49], are the complex permittivities of the amorphous (m=0) and crystalline (m=1.0) GST at wavelength λ, respectively.

## 3. Results and Discussion

### 3.1. Spectral and Field Characteristics of ED-SLRs

**Figure 2:**
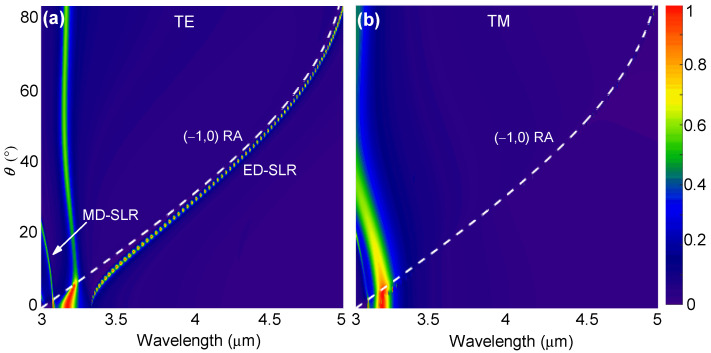
Calculated dispersion of the ED-SLR of the GST metasurface with m=0. The reflectance spectra as a function of the incidence angle were simulated for (**a**) TE- and (**b**) TM-polarized incident light. White dashed curves represent the RA of the (−1,0) diffraction order.

Figure 2 depicts the simulated reflectance spectra of the GST metasurface in the amorphous state (m=0) and under TE- or TM-polarized plane wave incidence at different angles. The white dashed curves indicate the RA curves of the (p,q) diffraction orders, which are determined by

(2) $$\begin{array}{c} 1=\sqrt{{\left(\sin\theta +p\frac{\lambda_{\mathrm{RA},(p,q)}\left(\theta \right)}{n_{0}\Lambda }\right)}^{2}+{\left(q\frac{\lambda_{\mathrm{RA},(p,q)}\left(\theta \right)}{n_{0}\Lambda }\right)}^{2}}\; \end{array}$$

For the TE polarization, Figure 2a shows that extremely narrow reflection peaks can be observed near the (−1,0) order RA curve. As the incidence angle increases, these peaks converge with the RA curve. Meanwhile, there exists another narrow reflection curve with relatively small peak values on the leftmost side. For the TM polarization, however, similar narrow peaks can hardly be observed under oblique incidence, as shown by Figure 2b. These dispersion behaviors are very similar to Figure 2 in [15], and thus we surmise that they should have the same underlying physics: for TE polarization, the dispersion band near the (−1,0) RA should correspond to the ED-SLR, whereas the leftmost band should correspond to the MD-SLR; for TM polarization, no MD-SLR can be observed for wavelengths that are larger than $\lambda_{\mathrm{RA},(0,\pm 1)}\left(\theta =0\right)$, which equals to $n_{0}\Lambda =3$ μm here. Therefore, hereafter, we will restrict ourselves to the TE polarization since we focus on the mid-infrared regime in this work.

**Figure 3:**
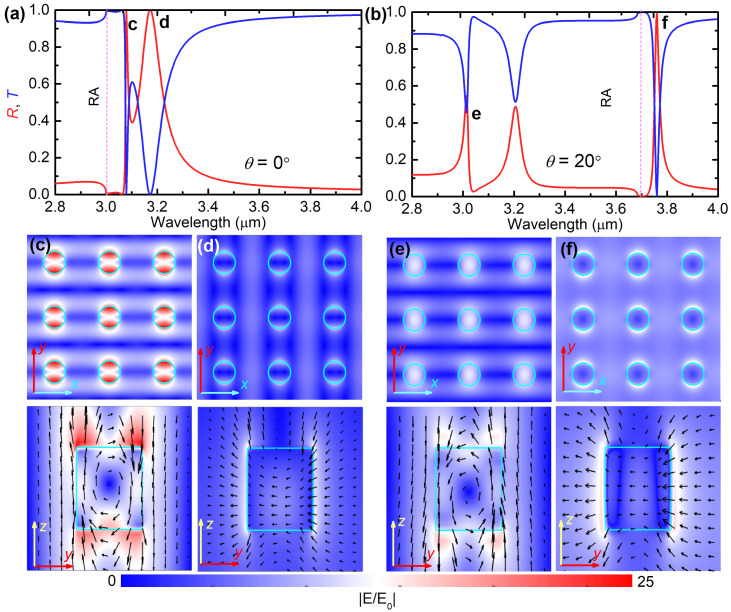
(**a**,**b**) Simulated reflectance (red) and transmittance (blue) spectra for TE-polarized incident light at (**a**) normal incidence ($\theta =0^{\circ }$) and (**b**) oblique incidence of angle $\theta =20^{\circ }$. Vertical pink dashed lines represent the (−1,0) order RA wavelengths. (**c**,**d**) near-field electric field distributions normalized to the incident field $E_{0}$, $|E/E_{0}|$ showing the xy- and yz-planes intersecting the nanoparticle at its center, for (**c**) λ=3.081 μm and (**d**) λ=3.171 μm at $\theta =0^{\circ }$, and (**e**) λ=3.013 μm and (**f**) λ=3.759 μm at $\theta =20^{\circ }$. Color scale represents the electric field intensity, and arrows indicate the electric field directions.

In Figure 3a,b, we re-plot the reflectance, transmittance, and absorbance spectra for the normal incidence ($\theta =0^{\circ }$) and for the oblique incidence of a typical angle $\theta =20^{\circ }$. Under normal incidence, we observed two reflectance peaks, and correspondingly two transmittance dips on the right side of the RA wavelength (indicated by the vertical pink dashed line): A very sharp one locating at λ=3.081 μm, and a stronger resonance at λ=3.171 μm. These spectral features are similar to the results in [9] (Figure 2c with d=110 nm) and [15] (Figure 3a), and thus should attribute to the same origin: the first and the second resonances should be assigned to the MD-SLR and the ED-SLR, respectively. In order to confirm this assignment, we simulate the spatial distribution of the electric fields at these two wavelengths. Figure 3c shows that, at λ=3.081 μm, the electric field shows a circulation inside the nanorod, strong intensity enhancement, and extended mode volume over the entire array, which are characteristics of the MD-SLR [9,15]. However, at λ=3.171 μm, the electric field in Figure 3d is greatly enhanced at the vertical edges of the nanoparticle, is aligned with the incident field showing a dipolar field pattern, and is extended outside the nanoparticles with large intensities. These are typical signatures of the ED-SLR [9,15].

Under the oblique incidence at angle $\theta =20^{\circ }$, Figure 3b shows that there exist three reflectance peaks (or transmittance dips). Among these, the first and the third one locating at λ=3.013 μm and λ=3.759 μm, respectively, are narrow and have a Fano-shaped profile. The simulated electric field distributions for these two wavelengths are depicted in Figure 3e,f. At λ=3.013 μm, Figure 3e shows that the simulated electric fields have similar distributions as Figure 3c. Therefore, we assign the first narrow band in Figure 2a to the MD-SLR. Compared with the results under normal incidence, the electric field enhancement under the oblique incidence at angle $\theta =20^{\circ }$ is weaker, corresponding to the wider linewidth of the MD-SLR. At λ=3.759 μm, however, Figure 3f shows similar characteristics as those in Figure 3c, confirming the excitation of the ED-SLR. Compared with the results under normal incidence in Figure 3c, the electric field enhancement under the oblique incidence at angle $\theta =20^{\circ }$ is stronger, corresponding to the narrower linewidth of the ED-SLR.

### 3.2. Effects of the Geometric Size

**Figure 4:**
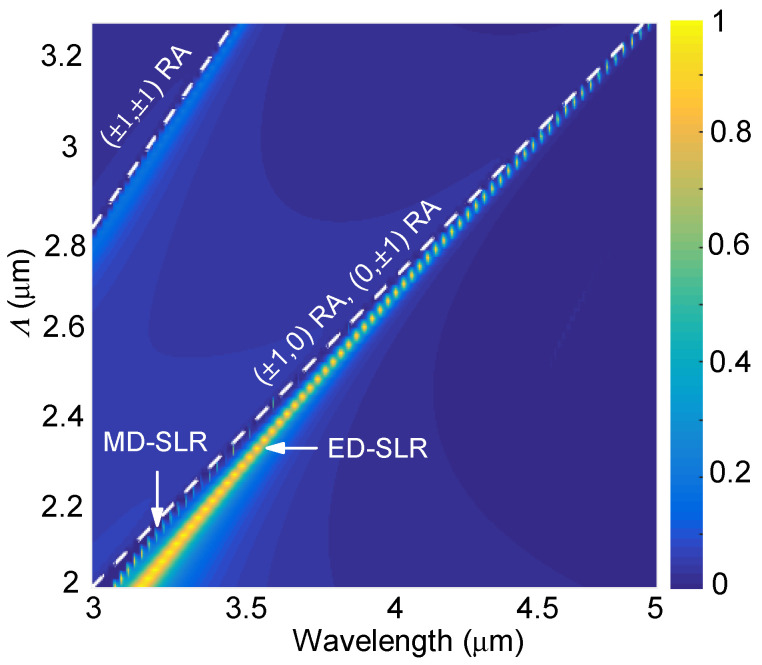
Simulated reflectance spectra for different periods. Note that, under normal incidence, RAs of the (±1,0) and (0,±1) orders degenerate into one curve.

We now investigate the effects of the geometric size on the ED-SLR and MD-SLR obtained under normal incidence. The calculations were performed under normal incidence with TE-polarization. Note that, under normal incidence, TM and TE polarizations share the same results due to the symmetry, Figure 4 shows that there exist two branches of reflection peaks: an extremely narrow one and a relatively wider one. As the metasurface period increases from Λ=2 μm to 3.3 μm, the narrower branch converges faster with the RA line of the (±1,0) or (0,±1) order compared with the wider branch. These behaviors further confirm the Mie SLR effect: the narrower and wider branches correspond to the MD-SLR and the ED-SLR, respectively.

**Figure 5:**
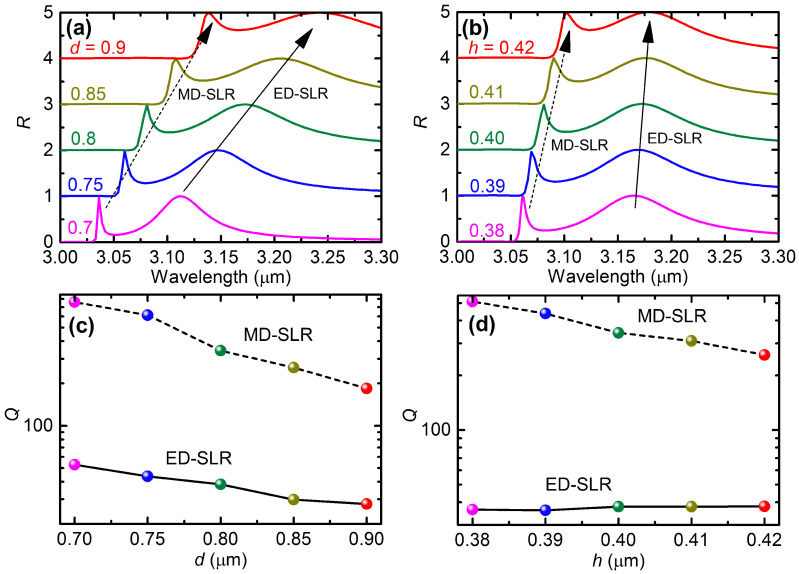
(**a**,**b**) Simulated reflectance spectra and (**c**,**d**) the associated quality factors of ED-SLR and MD-SLR for different (**a**,**c**) diameters and (**b**,**d**) heights (the values of both parameters are in units of μm).

As the nanorod diameter increases from 0.7 μm to 0.9 μm, Figure 5a shows that both the MD-SLR and the ED-SLR are both red-shifted by ∼0.115 μm. The spectrum of the MD-SLR is broadened significantly, whereas that of the ED-SLR is broadened slightly. Correspondingly, the quality factor decreases dramatically from Q=759 to 185 for the MD-SLR, but slightly from Q=53 to 28 for the ED-SLR, as shown by Figure 5c. On the other hand, as the nanorod height increases from 0.38 μm to 0.42 μm, Figure 5b,d show that similar behaviors can be observed. The exception is that both the redshifts and the decrease of the quality factors are much smaller.

Therefore, the small decrease of the ED-SLR quality factor suggests large fabrication tolerance on both the diameter and the height of the GST nanorods in practical fabrication based on e-beam lithography and reactive ion etching, during which the period can be accurately controlled. These characteristics will greatly facilitate the nanofabrication of the proposed metasurface.

### 3.3. Large Spectral Tunability via Varying the Incidence Angle

**Figure 6:**
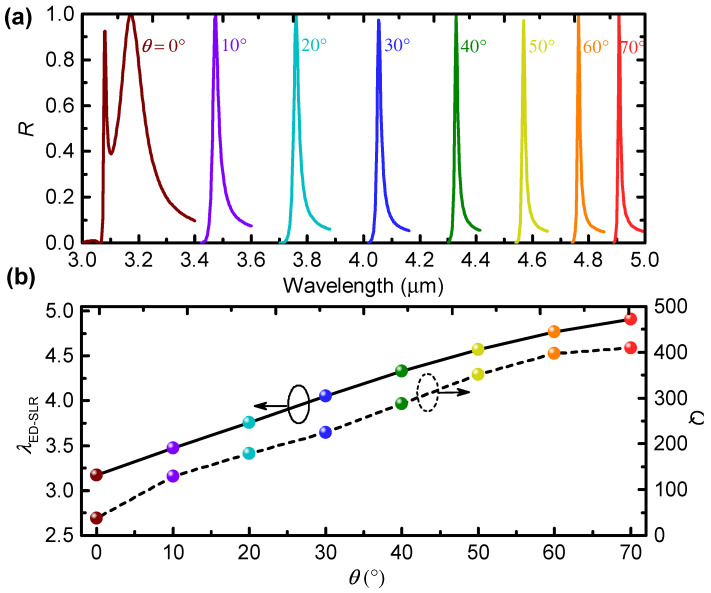
(**a**) Simulated reflectance spectra for different incidence angles. For clarity, the tails are not shown; (**b**) the resonance wavelength and the quality factor of the ED-SLR as a function of the incidence angle.

In Figure 2a, we also find that the reflectance peaks, which converge with the RA line, narrow down as the incidence angle increases. This behavior is better visualized by Figure 6a, where the tails of the reflectance spectra are not shown for clarity. Remarkably, the peak reflectance at resonance is always close to unity, indicating excellent filtering performance in terms of the insertion loss. This is because, in the amorphous state, GST has negligible absorption loss in the mid-infrared regime [16,21,22,23].

We extract the resonance wavelength and the quality factor of the ED-SLRs, and plot these results as a function of the incidence angle in Figure 6b. Strikingly, both the resonance wavelength and the quality factor increase almost linearly with the incidence angle. As θ increases $0^{\circ }$ to $70^{\circ }$, the resonance wavelength of the ED-SLR can be tuned from 3.171 μm to 4.908 μm, covering almost the entire mid-infrared regime (3–5 μm). Meanwhile, the quality factor increases by more than ten times, from Q=38 for $\theta =0^{\circ }$ to Q=409 (corresponding to linewidth of 12 nm) for $\theta =70^{\circ }$. Note that the obtained quality factor of Q=409 is the highest for ED-SLRs in all-dielectric metasurfaces [8,9,10,11,12,13,14,15,45].

The increasing quality factor for larger incidence angle can be explained by the detuning between the localized Mie resonances and the RA, which is measured by

(3) $$\begin{array}{c} \Delta =\lambda_{\mathrm{RA},(-1,0)}\left(\theta \right)-\lambda_{\mathrm{Mie},\mathrm{EDR}}\;. \end{array}$$

Here, $\lambda_{\mathrm{Mie},\mathrm{EDR}}=2.666$ μm is the localized Mie EDR of a single nanorod and is obtained according to the Mie theory calculations. Combining Figure 2 and Equations (2) and (3), we find that the quality factor increases almost linearly with the detuning. This is because, as the detuning increases, the interaction between the localized Mie EDR and the RA becomes weaker, leading to narrower ED-SLRs with more diffractive fields as shown by Figure 3d,f. These behaviors are consistent with the literature on Mie SLRs [15], as well as on plasmonic SLRs [50,51].

Therefore, we have shown that, by varying the incidence angle, the resonance wavelength of the ED-SLR can be tuned over an extremely large spectral region covering almost the entire mid-infrared regime. By increasing the incidence angle, the quality factor of the ED-SLR can be improved linearly owing to the increasing detuning between the Mie EDR of a single nanorod. Under oblique incidence of angle $\theta =70^{\circ }$, the quality factor is more than ten times of that obtained under normal incidence. As a consequence, the optical response of the designed metasurface can be actively tuned to a desired wavelength in the mid-infrared region while keeping a narrow linewidth by changing the incidence angle.

### 3.4. Active Tuning via Changing the GST Crystalline

**Figure 7:**
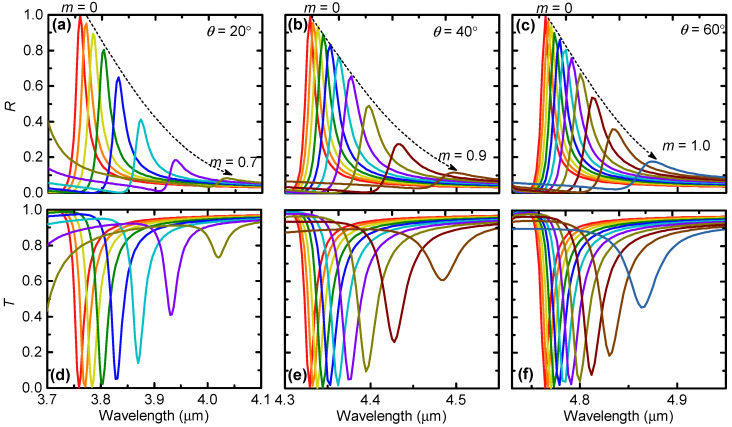
Simulated (**a**–**c**) reflection and (**d**–**f**) transmission spectra of the GST metasurface with different GST crystallization fractions varying from m=0 to a certain value when the reflection peak become less pronounced. The calculations were performed under TE-polarized incidence at three typical different oblique incidence angles, (**a**,**d**) $\theta =20^{\circ }$, (**b**,**e**) $\theta =40^{\circ }$, and (**c**,**f**) $\theta =60^{\circ }$.

While until now we have considered only the amorphous GST, hereafter, we study the effects of the GST crystallization fraction on the ED-SLR. Figure 7a,d show that, as *m* increases from 0 to 0.7, the narrowband reflectance peak or the transmittance dip obtained under the oblique incidence angle of $\theta =20^{\circ }$ is red-shifted from λ=3.759 μm to 4.032 μm. Meanwhile, the resonance is broadened and the peak reflectance decreases dramatically. Note that the results for m>0.7 are not shown for clarity since the reflectance peaks are too small in these cases.

For $\theta =40^{\circ }$, Figure 7b,e show that the dynamic wavelength tuning region becomes 4.329–4.497 μm when the GST crystallization fraction varies between 0≤m≤0.9. This dynamically tunable spectral range is smaller than that for $\theta =20^{\circ }$. As the incidence angle increases to $\theta =60^{\circ }$, the dynamically tunable spectral region further shifts to 4.767–4.875 μm, although, in this scenario, the reflectance peak is pronounced even for m=1. Therefore, as the incidence angle increases, the dynamically tunable spectral range keeps decreasing.

These results suggest that the proposed metasurface can act as a nonvolatile and reconfigurable narrowband bandpass filter in reflection and a bandstop filter in transmission in the meantime. Although the dynamical spectral tuning ranges are relatively small, the obtained quality factors are much larger than those of tunable filters based on other resonances in the literature, including the localized surface plasmons [25,26,27,29], plasmonic SLRs [30,31,32,33], extraordinary optical transmission [34,35,36], F–P resonances [37,38], and localized Mie resonances [39,40,41,42,43,44].

**Figure 8:**
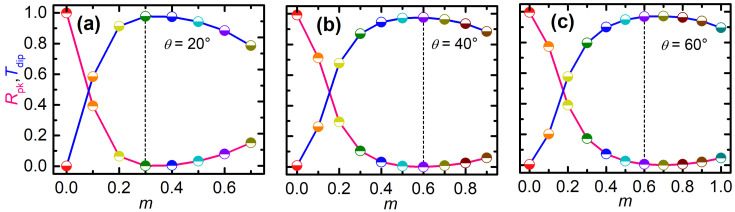
Peak reflectance (red curves) and dip transmittance (blue curve) for specific incidence angles and wavelengths as a function of the GST crystallization fraction. The results are extracted from Figure 7 for (**a**) $\theta =20^{\circ }$ and λ=3.759 μm, (**b**) $\theta =40^{\circ }$ and λ=4.329 μm, and (**c**) $\theta =60^{\circ }$ and λ=4.767 μm. The vertical dashed lines indicate the GST crystallization fraction for near-unitary peak reflectance or near-zero dip transmittance: (**a**) m=0.3 and (**b**,**c**) m=0.6.

We extract the values of peak reflectance and dip transmittance for different GST crystallization fractions at specific incidence angles and wavelengths, and plot the data in Figure 8. Figure 8a shows that, for $\theta =20^{\circ }$ and λ=3.759 μm, multilevel optical modulation in terms of the peak reflectance or dip transmittance can be obtained by using a small change of the GST crystalline (0≤m≤0.3). Note that, here λ=3.759 μm is the resonant wavelength for m=0, as indicated by the reflectance peak or the transmittance dip. In terms of the peak reflection or the dip transmission, the modulation depth is close to 100%. This suggests that the proposed metasurface in the two GST states of m=0 and m=0.3 can also function as a high-performance optical switch: the extinction ratio reaches as high as 24 dB for the reflection or 29 dB for the transmission, and the insertion loss is down to 0.005 dB for the reflection or 0.1 dB for the transmission. Since the GST crystallization fraction can be finely tuned using nanosecond [36] or femtosecond [42] optical pulses (for example, up to 10 states were demonstrated in [42]), these high-performance optical modulations or switching are all-optical, rapid, nonvolatile, and reversible. For other incidence angles and wavelengths such as ($\theta =40^{\circ }$, λ=4.329 μm) and ($\theta =60^{\circ }$, λ=4.767 μm), Figure 8b,c shows that similar high performance can be obtained, except that a larger change in the GST crystalline may be required.

Therefore, we have shown that the operation wavelength of the high-performance dynamically tunable optical filtering, modulation or switching can be conveniently tuned by varying the incidence angle.

## 4. Conclusions

In conclusion, we have numerically demonstrated extremely narrow and actively tunable ED-SLRs in GST metasurfaces. Simulation results have shown that the extremely narrow ED-SLR can be excited close to the (−1,0) order RA wavelength for the TE polarization under oblique incidence. The Mie SLR effects have been validated by the dispersion diagram, the near-field distributions, and the dependence on the period. We have also shown that the quality factor of the ED-SLR obtained under normal incidence decreases slightly as the nanorod diameter or height increases, suggesting a large fabrication tolerance. As the incidence angle increases from $\theta =0^{\circ }$ to $70^{\circ }$, the quality factor of the excited ED-SLR is enhanced by more than ten times, from Q=38 to Q=409, and that the angular dependent spectral tuning region can cover almost the entire mid-infrared regime. We have attributed the greatly increased quality factor to the weaker interaction between the localized Mie EDR and the RA due to the larger detuning. By changing the GST crystallization fraction, we have also observed red-shifts of the ED-SLR with decreasing peak reflectance or dip transmittance. Making use of this effect, we have numerically illustrated several promising applications, including reconfigurable narrowband optical bandpass or bandstop filters with high quality factors, multilevel all-optical modulation with modulation depth close to 100%, and all-optical switching with high extinction ratio and low insertion loss. Although in this work we have focused on the mid-infrared region, in which GST has relatively low absorption loss, the conclusions are applicable to the near-infrared and visible regions, where other phase-change materials such as Sb_2_S_3_ and Sb_2_Se_3_ suffer from much lower loss [19,20]. We therefore expect this work will advance the engineering of Mie SLRs, and the proposed GST metasurface will find potential applications in optical telecommunication, networks, and microsystems.

## Data Availability

The data presented in this study are available on request from the corresponding author.
